# Lulo cell line derived from *Lutzomyia longipalpis *(Diptera: Psychodidae): a novel model to assay *Leishmania *spp. and vector interaction

**DOI:** 10.1186/1756-3305-4-216

**Published:** 2011-11-14

**Authors:** Luzia MC Côrtes, Roger MM Silva, Bernardo AS Pereira, Camila Guerra, Angela C Zapata, Felio J Bello, Léa C Finkelstein, Maria F Madeira, Reginaldo P Brazil, Suzana Côrte-Real, Carlos R Alves

**Affiliations:** 1 Laboratório de Biologia Molecular e Doenças Endêmicas, Av. Brasil 4365, Rio de Janeiro - CEP 21040-360, Brasil; 2 Laboratório de Biologia Estrutural, Av. Brasil 4365, Rio de Janeiro - CEP 21040-360, Brasil; 3 Departamento de Ciencias Básicas, Universidad De La Salle, Carrera 2 No. 10-70, D.C., Colombia; 4 Universidad Del Rosário, Escuela de Medicina, Carrera 24 no 63C-69, Bogotá, D.C. Colombia; 5 Laboratório Imunoparasitologia - IOC - FIOCRUZ, Av. Brasil 4365, Rio de Janeiro - CEP 21040-360, Brasil; 6 Laboratório de Vigilância em Leishmanioses - IPEC - FIOCRUZ, Av. Brasil 4365, Rio de Janeiro - CEP 21040-360, Brasil; 7 Laboratório de Bioquímica e Fisiologia de Insetos, Av. Brasil 4365, Rio de Janeiro - CEP 21040-360, Brasil

**Keywords:** *Leishmania *spp, Lulo cell, *Phlebotominae*, promastigotes

## Abstract

**Background:**

*Leishmania (Vianna) braziliensis*, *Leishmania (Leishmania) amazonensis and Leishmania (Leishmania) chagasi *are important parasites in the scenario of leishmaniasis in Brazil. During the life cycle of these parasites, the promastigote forms adhere to the midgut epithelial microvillii of phlebotomine insects to avoid being secreted along with digestive products. Lulo cells are a potential model that will help to understand the features of this adhesion phenomenon. Here, we analyze the interaction between *Leishmania *spp. promastigotes and Lulo cells *in vitro*, specifically focusing on adhesion events occurring between three *Leishmania *species and this cell line.

**Methods:**

Confluent monolayers of Lulo cells were incubated with promastigotes and adhesion was assessed using both light microscopy and scanning electron microscopy.

**Findings:**

The results indicate that species from the subgenera *Leishmania *and *Viannia *have great potential to adhere to Lulo cells. The highest adherence rate was observed for *L. (L.) chagasi *after 24 h of incubation with Lulo cells (27.3 ± 1.8% of cells with adhered promastigotes), followed by *L. (L.) amazonensis *(16.0 ± 0.7%) and *L. (V.) braziliensis *(3.0 ± 0.7%), both after 48 h. In the ultrastructural analysis, promastigote adherence was also assessed by scanning electron microscopy, showing that, for parasites from both subgenera, adhesion occurs by both the body and the flagellum. The interaction of Lulo cells with *Leishmania (L.) chagasi *showed the participation of cytoplasmic projections from the former closely associating the parasites with the cells.

**Conclusions:**

We present evidence that Lulo cells can be useful in studies of insect-parasite interactions for *Leishmania *species.

## Findings

Leishmaniases are infections that affect humans, wildlife and domestic animals, and present an array of clinical manifestations, varying from tegumentary (mucocutaneous, cutaneous and diffuse) to visceral. Species of the genus *Leishmania *are present in all continents except Antarctica, and many of them are causative agents of disease. Recent literature indicates a sharp increase in the number of cases in developed non-endemic countries, pointing to a current escalation of 'imported leishmaniasis' cases [1]. In this epidemiologic context, *Leishmania (Vianna) braziliensis*, *Leishmania (Leishmania) amazonensis and Leishmania (Leishmania) chagasi *are the major causative agents of leishmaniasis in Brazil and, thus, are a significant public health issue [2].

During the life cycle of these parasites, they present two major morphological forms: extracellular promastigotes with visible flagellum, which multiply in the midgut of the sandfly vector, and intracellular non-motile amastigotes, that live within macrophages of the vertebrate host [3]. These parasites are transmitted to the mammalian host during the blood meal of infected sandfly vectors of the genus *Lutzomyia *(in the Americas). Sandflies inoculate extracellular promastigotes at the bite site, which are then phagocytosed by macrophages [4]. Complex interactions occur between *Leishmania *parasites and their sandfly vectors. *Leishmania *promastigotes live exclusively within the midgut of sandflies and attach to it using surface glycoconjugates, a key step in establishment of the infection. Differentiation of promastigotes to mammal-infective stages is characterized by significant biochemical and cellular remodeling [4]. *Leishmania *spp. differentiation, maturation and replication *in vitro *has been achieved in cultures of human macrophage cells [5,6]; J774 (murine) macrophages [7,8]; fibroblast cell lines [9,10], epithelial cells [11], dendritic cells [12,13], neutrophil granulocytes [14]; *Aedes albopictus *cells [15], and *Aedes aegypti *cells [16,17], however, there are few studies about the life cycle of *Leishmania *promastigotes in phlebotomine sandflies which use insect cell lines [18,19].

Previous studies describe the establishment and characterization of a continuous cell line from *Lutzomyia longipalpis*, designated Lulo. These studies, demonstrated the susceptibility of this cell line to infections with arboviruses and *Leishmania (Leishmania) chagasi *[18,20]. The Lulo cell line is composed of epitheloid cells, originated from *Lu. longipalpis *embryonic tissue, that was obtained from adult insects collected and colonized in Colombia. The morphological, cytogenetical and biochemical characteristics of this cell line have already been studied [18]. The aim of this study is to emphasize new aspects in events of adhesion of three *Leishmania *species to cells from the Lulo cell line.

### Adhesion assays

In the present study, promastigotes of *L. (L.) amazonensis *(MHOM/BR/77/LTB0016), *L. (V.) braziliensis *(MCAN/BR/1998/619) and *L. (L.) chagasi *(MCAN/BR/2008/1112) were used. Parasites (10^6 ^cells/mL) were grown in BHI medium supplemented with 10% fetal calf serum (FCS) and maintained at 28°C. The insect epithelioid cell line Lulo was cultured in a 1:1 mix of L15 and Grace media supplemented with 10% FCS, penicillin (100 U/mL) and streptomycin (100 ug/mL), incubated at 28°C. Confluent monolayers were seeded on glass coverlips inside wells of a 24 well plate, to a final number of 2 × 10^5 ^cells per well, prior to interaction with parasites.

For kinetic studies of Lulo cells and promastigote interactions, a ratio of about 10:1 parasites/cells was used. Two hours after co-incubation non-adhered promastigotes were removed by three washing cycles with phosphate-buffered saline (PBS) pH 7.2 and cell cultures were followed for 24, 48 and 72 hours. The attached cells were fixed with methanol and stained with Giemsa.

For scanning electron microscopy assays, coverslips containing Lulo cells and parasites were incubated (1 h, 25°C) with 0.1 M sodium cacodylate buffer pH 7.2 containing 2.5% glutaraldehyde and 3.5% sucrose, followed by a second incubation (1 h, 25°C) with 1% osmium tetroxide and dehydrated in acetone. Afterwards, coverslips were dried with CO_2 _in a critical point dryer and coated with gold. Samples were examined in a scanning electron microscope (Jeol JSM-6390LV).

### Quantification and visualization of adhesion

**Figure 1:**
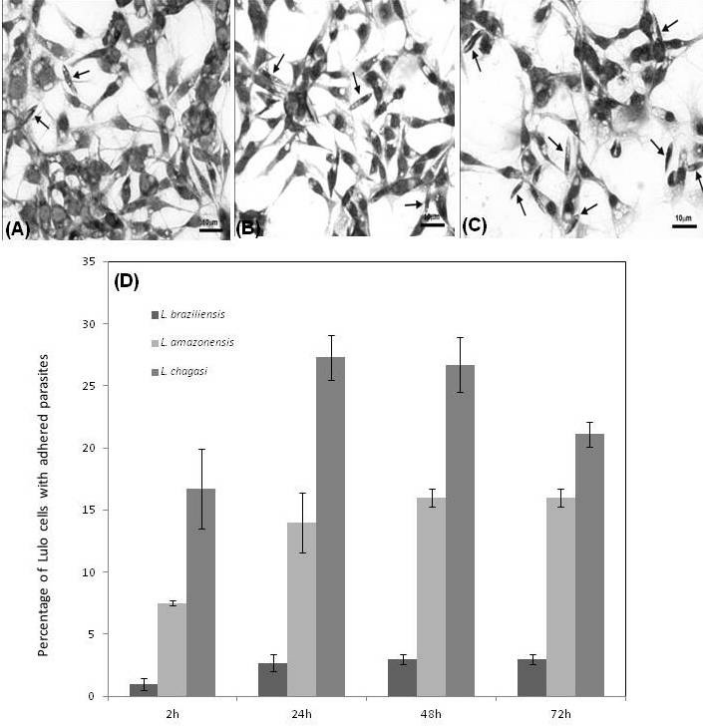
**Analysis of promastigote adhesion to Lulo cells**. After interaction between Lulo cells and promastigotes, the samples were stained with Giemsa for analysis. In the figure, the arrows are indicating the promastigotes adhered to Lulo cells at 48 h for *Leishmania (V.) braziliensis *(A), *Leishmania (L.) amazonensis *(B) and 24 h for *Leishmania (L.) chagasi *(C). Quantitative data (D) of the interaction were assessed at different times (2 h; 24 h; 48 h; 72 h) of incubation. Data are expressed in percentile values (%) and represent average and standard deviation of five independent experiments.

The optical microscopy analysis indicates a greater potential of species from the subgenus *Leishmania *to adhere to Lulo cells when compared to parasites from the subgenus *Viannia *(Figure 1). *L. (L.) chagasi *has the highest adhesion rate to Lulo cells (between 2 h to 72 h of interaction), followed respectively by *L. (L.) amazonensis *and *L. (V.) braziliensis*. Additionally, results point to differences with statistical significance for the adhesion rates (between 2 h to 72 h of interaction) of *L. (V.) braziliensis *× *L. (L.) amazonensis *(P = 0.010), *L. (V.) braziliensis *× *L. (L.) chagasi *(p = 0.020), *L. (L.) chagasi *× *L. (V.) braziliensis *(p = 0.020) and *L. (L.) chagasi *× *L. (L.) amazonensis *(p = 0.003). Peaks of adhesion were observed after 24 h of incubation with Lulo cells for *L. (L.) chagasi *(27.3 ± 1.8% of cells with adhered promastigotes) and 48 h for *L. (L.) amazonensis *(16.0 ± 0.7%) and *L. (V.) braziliensis *(3 ± 0.7%), (Figure 1).

The fact that *L. (V) braziliensis *showed a lower adhesion rate to Lulo cells when compared to *L. (L) amazonensis *and *L. (L) chagasi *is an interesting and unexpected finding: as *Lu. longipalpis *is reported as an efficient vector for species of the subgenera *Leishmani*a, like *L. (L) chagasi *[21], and as Lulo cells are derived from this sand fly species, it was reasonable to suppose that these cells would be more susceptible to adherence to parasites from the subgenera *Leishmania*. Thus, further studies are required to identify and understand the molecules related to promastigote adhesion on the surface of Lulo cells. Moreover, these results suggest that the Lulo cell line can be applied to studies of insect-parasite interactions for both tegumentary and visceral *Leishmania *species.

The present results suggest that this model of interaction could, at some level, be due to the strong adhesion observed between parasites and Lulo cells, which mimic the events that take place in the digestive tract of infected insects. Differences between adhesion rates of the three studied *Leishmania *species may indicate the presence of specific and distinct molecules involved in this process for each species [22].

**Figure 2:**
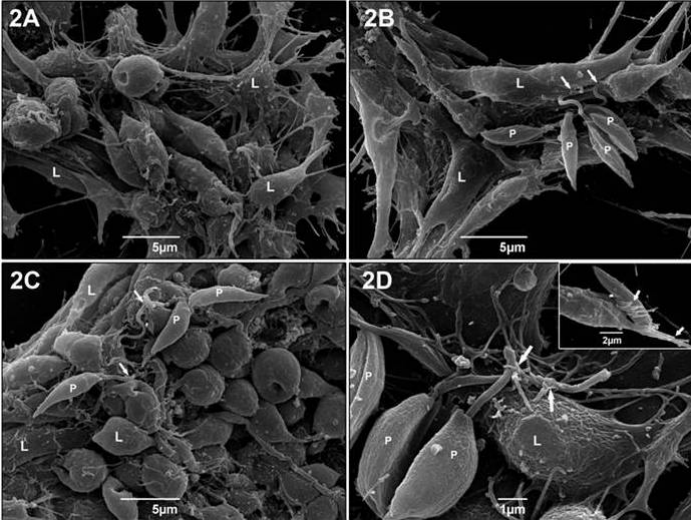
**Scanning electron microscopy showing the interaction of promastigotes and Lulo cells**. Electron micrographs show adhesion of parasites from three *Leishmania *species (P) with Lulo cells (L). **A **- Lulo cells; *in vitro*, present rounded morphology or sprawled with cytoplasmic projections. **B - ***Leishmania (V.) braziliensis; *adhesion occurs by flagellum (arrows) with tenuous contact between both cells and parasite. **C-D **- Interaction of species from the subgenus *Leishmania *with Lulo cells; occurs by both flagellum and body. **C**- *Leishmania (L.) amazonensis; *promastigotes are adhered by both body and flagellum (arrows) to cytoplasmic membrane of Lulo cells. **D **- Interaction of Lulo cells with *Leishmania (L.) chagasi; *occurs with participation of cytoplasmic projections involving the flagellum (arrows). In detail, we show flagellum (arrows) and body (arrows) of parasites that are being enveloped by several cytoplasmic projections of Lulo cells - **inset - D**. Images were assessed after 24 h (D) and 48 h (B and C) of interaction.

Scanning electron microscopy analysis showed that Lulo cells, when cultured *in vitro*, have either a rounded morphology or become sprawled with cytoplasmic processes (Figure 2a). Adhesion of promastigotes from the three studied *Leishmania *species to Lulo cells can occur by flagellum only or simultaneously by body and flagellum (Figure 2b-d). On the other hand, the interaction of Lulo cells with *L. (L.) chagasi *showed the participation of cytoplasmic projections, which closely associated the parasites to the cells (Figure 2-d).

In the invertebrate host, adhesion of promastigotes to gut epithelium is an essential step for the maintenance of the life cycle. Adhesion of parasites is directly related to the expression of membrane molecules, such as phosphoglycans and glycoproteins [23-30], distributed along their surface. Although there are currently a variety of cell lines and cell free media available to study the transformations occurring during the life cycle of different *Leishmania *species in mammals, there is little information regarding this process in cell cultures obtained from phlebotomine sandfly vectors.

## Conclusion

We have presented evidence that the Lulo cell line can be useful as a model for studies of insect-parasite interactions for *Leishmania *species. Further experiments that can bring significant contributions on understanding the biological cycle of these parasites during infection of insect cells are still necessary. Our present perspectives are targeted at confirming the potential of this model as an appropriate biological tool for studies of cell interactions with *Leishmania *spp.

## Competing interests

The authors declare that they have no competing interests.

## Authors' contributions

LMCC, RPB and CRA formulated the idea and wrote the manuscript; LMCC, ACZ, RMMS, CG, LCF and MFM performed the experimental processes. BASP, SCR and FJB provided critical comments to the protocol and the discussion. All authors approved the final version of this manuscript.

## References

[B1] Pavli A Maltezou HC Leishmaniasis, an emerging infection in travellers Int J Infect Dis 2010 14 e1032 9 10.1016/j.ijid.2010.06.019 20952234

[B2] Lainson R The Neotropical Leishmania species: a brief historical review of their discovery, ecology and taxonomy Rev Pan-Amaz Saude 2010 1 13 32

[B3] Bates PA Rogers ME New insights into the developmental biology and transmission mechanisms of Leishmania Curr Mol Med 2004 4 601 609 10.2174/1566524043360285 15357211

[B4] Bates PA Leishmania sand fly interaction: progress and challenges Curr Opin Microbiol 2008 11 340 344 10.1016/j.mib.2008.06.003 18625337 PMC2675783

[B5] Gantt KR Goldman TL McCormick ML Miller MA Jeronimo SMB Nascimento ET Britigan BE Wilson ME Oxidative responses of human and murine macrophages during phagocytosis of *Leishmania chagasi* J Immunol 2001 167 893 901 11441096 10.4049/jimmunol.167.2.893

[B6] Silva SdeO Wu AA Evans DA Vieira LQ Melo MN *Leishmania *sp isolated from human cases of cutaneous leishmaniasis in Brazil characterized as *Leishmania major*-like Acta Trop 2009 112 239 248 10.1016/j.actatropica.2009.07.026 19660430

[B7] Chang KP Reed SG McGwire BS Soong L *Leishmania *model for microbial virulence: the relevance of parasite multiplication and pathoantigenicity Acta Trop 2003 85 375 390 10.1016/S0001-706X(02)00238-3 12659975

[B8] Wanderley JL Pinto da Silva LH Deolindo P Soong L Borges VW Prates DB de Souza AP Barral A Balanco JM do Nascimento MT Saraiva EM Barcinski MA Cooperation between apoptotic and viable metacyclics enhances the pathogenesis of leishmaniasis PLoS One 2009 4 e5733 10.1371/journal.pone.0005733 19478944 PMC2684641

[B9] Corte-Real S Santos CB Meiralles MNL Differential expression of the plasma membrane Mg^2+ ^ATPase and Ca^2+ ^ATPase activity during adhesion and interiorization of *Leishmania amazonensis *in fibroblasts *in vitro* J Submicros Cytol Parasitol Pathol 1995 27 359 366 7671216

[B10] Hespanhol RC de Nazaré C Soeiro M Meuser MB de Nazareth SL Meirelles M Corte-Real S The expression of mannose receptors in skin fibroblast and their involvement in *Leishmania (L.) amazonensis *invasion J Histochem Cytochem 2005 53 35 44 15637336 10.1177/002215540505300105

[B11] Pessotti JH Zaverucha Do Valle T Corte-Real S Conçalves da Costa SC Interaction of *Leishmania (L.) chagasi *with the vero cell line Parasite 2004 11 99 102 15071834

[B12] Jebbari H Stagg AJ Davidson RN Knight SC *Leishmania major *promastigotes inhibit dendritic cell motility in vitro Infect Immun 2002 70 1023 1026 10.1128/IAI.70.2.1023-1026.2002 11796645 PMC127657

[B13] Colmenares M Corbi AL Turco SJ Rivas J The dendritic cells receptor DC-SIGN discriminates among species and life cycle forms of *Leishmania* J Immunol 2004 172 1186 1190 14707095 10.4049/jimmunol.172.2.1186

[B14] Laskay T Zandbergen G Slobach W Neutrophil granulocytes - Trojan horses for *Leishmania major *and other intracellular microbes? Trends Microbiol 2003 11 210 214 10.1016/S0966-842X(03)00075-1 12781523

[B15] Dedet JP Gaudin OG *Leishmania donovani *multiplication in a cell line *Aedes albopictus* Trans R Soc Trop Med Hyg 1976 70 535 536 841664 10.1016/0035-9203(76)90154-1

[B16] Miranda AA Sarmiento L Caldas ML Zapata C Bello FJ Morphology and cytochemistry of *Aedes aegypti's *cell cultures (Diptera: Culicidae) and susceptibility to *Leishmania panamensis *(Kinetoplastida: Trypanosomatidae) Rev Biol Trop 2008 56 447 458 19256419

[B17] Münoz-Camargo C Barreto A Bello F Análisis de la susceptibilidad de una línea celular de Aedes aegypti (Diptera: Culicidae) a la infección com *Leishmania (L) chagasi *y *Leishmania (V) braziliensis* Rev Cienc Salud Bogotá (Colombia) 2005 3 119 128

[B18] Rey G Ferro C Bello F Establishment and characterization of a new continuous cell line from *Lutzomyia longipalpis *(Diptera: Psychodidae) and its susceptibility to infections with arboviruses and *Leishmania chagasi* Mem Inst Oswaldo Cruz 2000 95 103 110 10.1590/S0074-02762000000100017 10656714

[B19] Zapata Lesmes AC Cárdenas Castro E Bello F Characterization of cell cultures derived from *Lutzomyia spinicrassa *(Diptera: Psychodidae) and their susceptibility to infection with *Leishmania (Vianna) braziliensis* Med Sci Monit 2005 11 457 464 16319783

[B20] Bello FJ Mejía AJ Corena MP Ayala M Sarmiento L Zúñiga C Palau MT Experimental infection of *Leishmania (L.) chagasi *in a cell line derived from *Lutzomyia longipalpis *(Diptera: Psychodidae) Mem Inst Oswaldo Cruz 2005 100 619 625 10.1590/S0074-02762005000600004 16302061

[B21] Killick-Kendrick R Phlebotomine vectors of the leishmaniases: a review Med Vet Entomol 1990 4 1 24 10.1111/j.1365-2915.1990.tb00255.x 2132963

[B22] Alves CR Côrtes LMC Brazil RP The vectorial potential of *Lutzomyia (Nyssomyia) intermedia *and *Lutzomyia (N.) whitmani *in the transmission of *Leishmania (V.) braziliensis *can also be related to proteins attaching J Biomed Biotechnol 2010 2010 827851 20589075 10.1155/2010/827851 PMC2879554

[B23] Saraiva EM Pimenta PF Brodin TN Rowton E Modi GB Sacks DL Changes in lipophosphoglycan and gene expression associated with the development of *Leishmania major *in *Phlebotumus papatasi* Parasitology 1995 111 275 287 10.1017/S003118200008183X 7567096

[B24] Sacks DL Hieny S Sher A Identification of cell surface carbohydrate and antigenic chances between noninfective and infective developmental stages of Leishmania major promastigotes J Immunol 1985 135 564 569 2582050

[B25] Sacks DL Saraiva EM Rowton E Turco SJ Pimenta PF The role of the lipophosphoglycan of leishmania in vector competente Parasitology 1994 108 suppl s55 62 8084656 10.1017/s0031182000075727

[B26] Pimenta PF Saraiva EM Sacks DL The comparative fine structure and surface glycoconjugate expression of three life stages of *Leishmania major* Exp Parasitol 1991 72 191 204 10.1016/0014-4894(91)90137-L 2009923

[B27] Sacks DL Kamhawi S Molecular aspects of parasite-vector and vector-host interactions in leishmaniosis Am Rev Microbiol 2001 55 453 483 10.1146/annurev.micro.55.1.453 11544364

[B28] Fampa P Correa-da-Silva MS Oliveira SM Motta MC Saraiva EM Interaction of insect trypanosomatids with mosquitoes, sans fly and the respective insect cell lines Int J Parasitol 2003 33 1019 1026 10.1016/S0020-7519(03)00124-3 13129523

[B29] Novozhilova NM Bovin NV Structure, functions, and biosynthesis of glycoconjugates of Leishmania spp. cell surface Biochemistry 2010 75 686 694 10.1134/S0006297910060027 20636259

[B30] Myskova J Svobodava M Beverley SM Volf P A lipophosphoglycan-independent development of leishmania in permissive sand flies Microbes and infection 2007 9 317 324 10.1016/j.micinf.2006.12.010 17307009 PMC2839925

